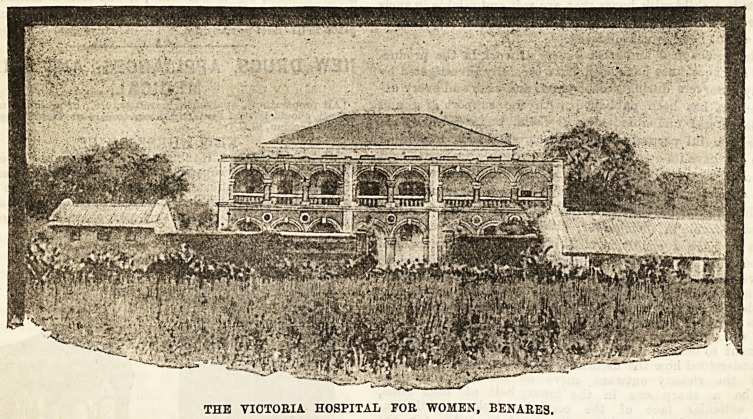# The Woman Question in India

**Published:** 1891-09-26

**Authors:** 


### The Woman Question in India.

A short time ago there was published a little book which gave an account of the marriage of a Bombay merchant with an Indian widow. We are all quite familiar with the fact that in India mere children are wives, that many such children inevitably become widows, and that for a man to marry one of such widowed children or grown up women is for him to degrade and disgrace himself for ever in the eyes of his fellow-countrymen. The plain fact, without any exaggeration, is, that women in India exist and are used merely for the convenience of men. They are shut up in their zenanas, and have no practical contact with the intelligence, moral influence, and freedom of the outer world whatsoever. Now it is in every way a wrong, foolish, and most unhappy state of things which reduces intelligent human beings to such a level. One cannot but feel angry with the stupidity and grossness of the male part of the population who have instituted such a system, and with the female part who have submitted to it. Reason forces upon us a conclusion that we who are better instructed ought to take practical steps to put an end to such things. Religion, the Christian religion at any rate, insists that women have souls to be saved or lost, as well as bodies to be maltreated; and that it would be wicked conduct in the sight of God to pass by the millions of Indian women who, through the fault of a bad social system, live and die in unutterable and hopeless ignorance. This country rules India, and one of its first duties, therefore, is to teach India those great principles of knowledge and conduct which have made it able to conquer and rule that vast empire. The Zenana Bible and Medical Mission has voluntarily taken upon itself the duties which the country ought to perform. It sends educated English women into the zenanas to teach the uneducated Indian women. It sends, above all, women doctors, to minister to the terrible bodily ills which come upon and remain with the Indian women as the result of their complete isolation from the outer world, and, therefore, from skilled medical help. The poor, helpless and untaught women of the zenanas are thus healed and comforted in body, and instructed and elevated in mind. Is it possible to imagine any kind of work which appeals more strongly and pitifully to the kindly human heart? Let any reader think what is the number of women who are shut out altogether from the great world in the different zenanas throughout India! There are about forty millions: more than the whole population of Great Britain. Twenty millions of these are widows, and to be a widow in India is to endure a lot more grievous than that of many English criminals. Of the twenty million widows seventy-nine thousand are child widows under nine years of age. No words of description can add to the pathos of these plain and simple facts. The Zenana Bible and Medical Mission does its best to overtake the almost impossible work that is set before it. There are some five hundred and eighty missionaries who devote their lives to a hundred and thirty-nine millions of Indian women, a number so few that the efforts they make seem almost like the efforts of despair. But there is no doubt whatever that the whole system of Indian civilisation as it affects women is destined to be revolutionised by the Zenana Missions and similar agencies. A hundred and fifty pounds a year will support a lady medical missionary in her work in the zenanas. We cannot too earnestly appeal to our readers to help forward this great work of delivering the millions of Indian widows and married women from their almost living death. The Zenana Bible and Medical Mission desires to raise its income to thirty thousand a year. Lord Kinnaird and Sir William Muir, the Treasurers, will thankfully receive contributions, as will also the Hon. Finance Secretary, W. T. Paton, Esq., 2, Adelphi Terrace, W.C. The General Secretary of the Mission is the Rev. A. R. Cavalier.

THE VICTORIA HOSPITAL FOR WOMEN, BENARES.

---

Every householder has painful experience of the difficulty of getting good coffee, and it is rather hard that the difficulty should be increased by the manufacture of artificial coffee beans. These beans, which are being manufactured on a large scale in America, are said to be composed of rye flour, glucose, and water. They are prepared to resemble in size and colour a moderately good sample of roasted coffee beans, and by the introduction of some genuine beans they acquire the aroma of true coffee. Though the manufacturers sell the article as coffee substitutes, it is alleged that retailers in the States either sell it as genuine or mix it with genuine coffee.

## Figures and Tables

**Figure f1:**